# Non-cultivated Cotton Species (*Gossypium* spp.) Act as a Reservoir for Cotton Leaf Curl Begomoviruses and Associated Satellites

**DOI:** 10.3390/plants8050127

**Published:** 2019-05-14

**Authors:** Sara Shakir, Syed Shan-e-Ali Zaidi, Atiq-ur-Rehman, Muhammad Farooq, Imran Amin, Jodi Scheffler, Brian Scheffler, Muhammad Shah Nawaz-ul-Rehman, Shahid Mansoor

**Affiliations:** 1 National Institute for Biotechnology and Genetic Engineering, 38000 Faisalabad, Pakistan; shakir.sara@yahoo.com (S.S.); Shan.e.ali@outlook.com (S.S.Z.); atiqkhan09@yahoo.com (A.-u.-R.); qadrikazmi@yahoo.com (M.F.); imranamin1@yahoo.com (I.A.); 2 Virology Lab, Centre for Agricultural Biochemistry and Biotechnology, University of Agriculture, 38000 Faisalabad, Pakistan; msnawazulrehman@uaf.edu.pk; 3 Crop Genetics Research Unit, USDA-ARS, 141 Experiment Station Rd, Stoneville, MS 38776, USA; Jodi.Scheffler@ars.usda.gov; 4 Genomics and Bioinformatics Research Unit, USDA-ARS, 141 Experiment Station Rd, Stoneville, MS 38776, USA; Brian.Scheffler@ars.usda.gov

**Keywords:** Alphasatellite, *Begomovirus*, Betasatellite, Cotton leaf curl disease, *Gossypium* spp.

## Abstract

A collection of cultivated and non-cultivated species of cotton (*Gossypium* spp.) has been maintained for the last four decades in Multan, Pakistan. This geographical location has been observed as a hotspot for the evolution of begomoviruses and satellites associated with cotton leaf curl disease (CLCuD). Recent studies showed that begomoviruses responsible for the CLCuD epidemic in the 1990s, and that almost disappeared from the CLCuD complex in 2000s, have been observed again in CLCuD-infected cotton fields. To identify host species that acted as probable reservoirs for these viruses, we characterized begomoviruses and satellites in non-cultivated cotton species *G. raimondii*, *G. thurberi* and *G. mustelinum* and identified several species of CLCuD associated begomoviruses and satellites. Further, phylogenetic analysis indicated that the identified begomoviruses and beta/alphasatellites are closely related to the ones associated with the most recent CLCuD complex. qPCR indicated that the comparative level of virus significantly decreased in the presence of alphasatellites. Our results indicated that non-cultivated cotton species have been continuously challenged by diverse begomoviruses and associated satellites and act as reservoirs for CLCuD associated begomoviruses. These results provide novel insights into understanding the spread of begomoviruses and associated satellites in New World cotton species introduced into the Old World.

## 1. Introduction

Cotton is the world’s largest fiber producing crop and determines the economy of major producers like Pakistan and India [1]. However, cotton leaf curl disease (CLCuD) is the major constraint to cotton production in the Indian subcontinent [2]. CLCuD is caused by single stranded DNA (ssDNA) viruses belonging to the genus *Begomovirus* (family Geminiviridae) of plant viruses, that are transmitted via the insect vector, whitefly (*Bemisia tabaci*) [3].

Begomoviruses are classified into bipartite and monopartite; bipartite having two genomic components DNA-A and DNA-B, and monopartite having a single genomic component similar to the DNA-A of bipartite begomoviruses. DNA-A (genome size ~2.7 kb) of begomoviruses encodes the replication associated protein (Rep), replication enhancer protein (REn), transcriptional activator protein (TrAP), coat protein (CP), V2 and C4 proteins required for virus replication and gene expression. DNA-B (genome size ~2.7 kb) encodes a movement protein (MP) and nuclear shuttle protein (NSP) required for virus inter- and intra-cellular movement [4]. Although DNA-A and DNA-B have different genome organization, they share a very similar ‘common region’ (CR) with an exact stretch of nine nucleotides called nonanucleotide (TAATATT/AC). The part of CR spanning the nonanucleotide forms a stem-loop and provides the site for nicking and replication initiation [5].

Begomoviruses in the Old World (OW) are usually associated with ~1.4 kb subgenomic betasatellites, which determine the pathogenicity and symptom severity of the virus. Betasatellites encode a single βC1 protein and depend on their helper virus for their replication, encapsidation and movement [6]. Alphasatellites (genome size ~1.4 kb) are also associated with begomoviruses. They encode their own replication protein (alpha Rep) and are not involved in the pathogenicity of their helper *begomovirus*. Alpha Rep has also been reported to be a suppressor of post-transcriptional gene silencing (PTGS) [6,7]. Some begomoviruses have also been found associated with non-coding satellites (genome size ~630–750 bp) referred to as deltasatellites [8,9]. So far all begomoviruses that have been characterized and experimentally demonstrated to cause CLCuD are monopartite, and are associated with a specific betasatellite, cotton leaf curl Multan betasatellite (CLCuMuB) [10].

The *Gossypium* genus belongs to the family Malvaceae and includes 46 diploid, five true tetraploid and one purported tetraploid species [11,12]. All the diploid *Gossypium* species originated from a common ancestor and diversified into eight groups, from A-G and K [13]. All tetraploid cotton species are allotetraploid and originated as a result of interspecific hybridization between a *G. arboreum* like A genome and *G. raimondii* like D genome [13]. Diploid *G. arboreum* has been cultivated for fiber production in the Indian subcontinent for hundreds of years [14]. Tetraploid *G. hirsutum* was introduced to the Indian subcontinent in 1818 from Mexico and because of its high production, it covers 98% of the cultivated land area where *G. arboreum* was previously grown [15]. However, unlike *G. arboreum, G. hirsutum* was not resistant to the co-evolved plant pathogens such as CLCuD causing begomoviruses [16].

CLCuD was first observed in the 1960s, and the first epidemic emerged in 1986 in the Multan area of the Punjab province in Pakistan [17]. In the 1990s, the CLCuD had spread to all major cotton growing areas of Pakistan, and to some parts of India as well [18]. CLCuD during the 1990s was caused by a begomovirus–betasatellite complex comprising six major *begomovirus* species, cotton leaf curl Kokhran virus (CLCuKoV), cotton leaf curl Multan virus (CLCuMuV), cotton leaf curl Rajasthan virus (CLCuRaV), cotton leaf curl Alabad virus (CLCuAlV), tomato leaf curl Bangalore virus (ToLCBaV) and papaya leaf curl virus (PaLCuV) [19,20,21]. As for subgenomic betasatellites, CLCuD was associated with a single, cotton leaf curl Multan betasatellite (CLCuMuB) [22]. The characterization of begomoviruses at that time indicated that the “Multan strain” was the most virulent strain causing CLCuD [23]. Several CLCuD resistant cotton varieties were developed using breeding, and the disease was overcome by the late 1990s [24]. However, in the early 2000s, the virus symptoms again started to emerge in Burewala (an agricultural area near Multan), Pakistan [25]. This became the second epidemic of CLCuD, caused by a recombinant virus identified as cotton leaf curl Kokhran virus-Burewala strain (CLCuKoV-Bur). Recent changes in the CLCuD complex have lead scientists to predict a third epidemic of CLCuD in the Indian subcontinent [26,27,28]. During both the epidemics of CLCuD in the Indian subcontinent, native cotton *G. arboreum* remained symptomless and presented immunity to CLCuD where *G. hirsutum*, on the other hand, remained highly susceptible [29]. There are experimental evidences of CLCuD infection in *G. arboreum* as a result of grafting with CLCuD infected *G. hirsutum*, but it is still resistant under field conditions [30]. 

In Pakistan, a collection of non-cultivated and cultivated cotton species have been maintained for the last four decades at the Central Cotton Research Institute in Multan (Punjab, Pakistan). Non-cultivated cotton species are being maintained for breeding purposes as they could be a potential source of genetic resistance against biotic and abiotic stresses. This geographical location and the surrounding areas have been the center of CLCuD and vector whitefly infestation since the first epidemic of CLCuD in Pakistan in the 1990s. There is a frequent exchange of whiteflies among cultivated and non-cultivated cotton species, allowing virus transmission and maintenance in the non-cultivated species. Due to the distinct genotypes and perennial growth habit of non-cultivated species, they supposedly impose different selection pressure on the viruses they harbor as compared to annually grown cultivated cotton. Thus, a unique virus population is maintained in these plants via whitefly transmission within this herbarium [7]. We have previously tested several non-cultivated species using rolling circle amplification (RCA) followed by restriction-cloning-sequencing and identified several begomoviruses and associated satellites. In our previous work, we demonstrated that multiple begomoviruses associated with first epidemic in 1990s are back in cotton fields in Pakistan [26] and a recent study from India corroborates our findings and reported the re-emergence of CLCuMuV in cotton fields in India as well [27]. Since the non-cultivated cotton species act as reservoirs of distinct begomoviruses [31], we hypothesized that they may have some role in the re-emergence of CLCuD associated begomoviruses.

In this study, three non-cultivated cotton species *G. raimondii* (diploid D5), *G. thurberi* (diploid D1) and *G. mustelinum* (tetraploid AD4) were studied for the identification of begomoviruses and associated satellites. We characterized begomoviruses, betasatellites and alphasatellites, performed comprehensive phylogenetic analysis, and identified a close relationship with the recent cotton-infecting *begomovirus* complex from Pakistan and India [26,27]. We also considered the pioneer study that detected geminiviruses from non-cultivated cotton species by Nawaz-ul-Rehman et al. 2012 as a reference to study phylogenetic and recombination relationships. Thus, our findings give further insight into the possible re-emergence of cotton-infecting *begomovirus* and satellites. The importance of our findings with reference to recent literature is also discussed.

## 2. Results

**Figure 1:**
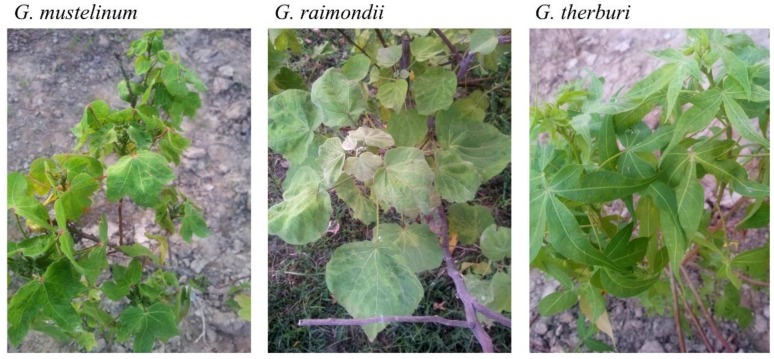
*Gossypium* species used in this study. *G. mustelinum, G. raimondii* and *G. thurberi* plants, maintained at the Central Cotton Research Institute (CCRI), Multan, Pakistan.

Non-cultivated species of cotton have been maintained in herbarium at CCRI Multan, Pakistan for the last four decades. Next generation sequencing (NGS) of RCA-enriched products detected diverse begomoviruses and associated satellites in tetraploid (AD) *G. mustelinum*, and diploid (D) *G. raimondii* and *G. thurberi* species (Figure 1).

### 2.1. Assembly and Analysis of NGS Data Identified Begomovirus and Alpha/Betasatellites in G. mustelinum, G. raimondii and G. thurberi

The Illumina MiSeq platform yielded 339,171, 301,034 and 877,391 number of reads from *G. mustelinum*, *G. raimondii* and *G. thurberi* samples, respectively. The trimmed reads had a length distribution of ~151 in all libraries. The contigs of size ~2.8 kb and ~1.4 kb were likely to be *begomovirus* and alpha/betasatellites, respectively, because of their corresponding genome sizes. A mixed approach of de novo and reference-based assembly was used for the assembly of *begomovirus*/alphasatellite/betasatellite genomes. For reference-guided assembly, a comprehensive list of begomoviruses and satellites was prepared based on the information available from ICTV (www.ictvonline.org). Selected contigs from assemblies were checked for the presence of putative open reading frames (ORF) using NCBI ORF finder (www.ncbi.nlm.nih.gov/gorf). All contigs of ~2.8 kb length had ORF and genetic arrangement typical of DNA-A and/or DNA-B components of begomoviruses (Tables S1 and S2).

Similarly, ORF analysis of ~1.4 kb contigs indicated the presence of two types of molecules, one with a single ORF in virion sense orientation, similar to alphasatellites and the other with a single ORF in complementary sense orientation, similar to betasatellites. BLASTp of amino acid sequences indicated that these isolates encode alpha Rep and βC1, respectively (Table S3).

### 2.2. Diverse Species and Strains of Begomovirus/Alphasatellite/Betasatellite are Maintained by Non-cultivated Cotton Species

Following the *begomovirus* species/strain demarcation criteria [32], BLASTn and SDT analysis of ~2.8 kb contigs identified five distinct virus strains belonging to three *begomovirus* species, cotton leaf curl Multan virus (CLCuMuV), cotton leaf curl Alabad virus (CLCuAlV) and bhendi yellow vein mosaic virus (BYVMV) in the non-cultivated cotton species under study (details provided in Table 1). CLCuMuV sequences isolated in this study showed maximum identity (94%–99%) with CLCuMuV isolates reported from previously reported non-cultivated cotton species from CCRI-Multan in 2012 (EU365616, EU384574) [31] and recently reported CLCuMuV sequences isolated from cultivated cotton fields from Punjab-Pakistan (KX656786-KX656788 and KX656795-KX656801) [26] and Punjab-India (KY120359-KY120361) [27]. Similarly, CLCuAlV showed 92%–95% sequence identity with six CLCuAlV sequences isolated from cultivated cotton fields from Vehari (KX656789-KX656794) and Multan and Lobatum strains (accessions no. EU365617, EU384575, FJ218485 and FJ210467) reported from non-cultivated cotton species from CCRI-Multan in the 2012 [31]. BLASTn analysis of BYVMV sequences showed close homology (92%–97%) with BYVMV sequences reported from India (FJ176235, AJ002451, etc.). Association of BYVMV with CLCuD complex is quite unusual but a significant number of sequencing reads from NGS confirms their presence in non-cultivated cotton species. Detailed SDT analysis for the Pakistan strain of CLCuMuV (Figure S1), Multan and Lobatum strain of CLCuAlV (Figure S2), and Thanagan and Pakistan strain of BYVMV (Figure S3), can be found online in supplementary data. Recombination analysis was done by RDP4 beta v 4.96 using different algorithms available so far and no recombination was detected in the sequences reported in this study.

We also detected the DNA-B component of bipartite begomoviruses, associated with CLCuAlV (Table 1). All DNA-B molecules (accession no. MH760444-MH760454) were 94%–99% identical to CLCuAlV sequences isolated from *G. mustelinum, G. punctatum, G. stocksi, G. somalense* and *G. davidsonii* in Pakistan (FJ218488-FJ218491, EU384577-EU284578). It must be mentioned here that in the literature, the name *Gossypium punctatum* mild leaf curl virus (GPMLCuV) has also been used for this virus, that later became CLCuAlV after the revised *begomovirus* taxonomic criteria [32]. In this manuscript, virus name CLCuAlV has been used to avoid ambiguity. These CLCuAlV DNA-B components showed 82%–86% sequence identity with Sri Lankan cassava mosaic virus (SLCMV), Indian cassava mosaic virus (ICMV) and *Jatropha curcas* mosaic virus (JCMV), suggesting its close relationship with these and other related virus species. This observation is consistent with previous findings [31]. 

Surprisingly, alphasatellites were only detected in tetraploid *G. mustelinum*, and not in diploid *G. thurberi* and *G raimondii*. In *G. mustelinum,* five distinct species of alphasatellites were detected namely, *Gossypium darwinii* symptomless alphasatellite (GDarSLA), *Gossypium davidsonii* symptomless alphasatellite (GDavSLA), ageratum yellow vein Singapore alphasatellite (AYVSA), cotton leaf curl Burewala alphasatellite (CLCuBuA) and papaya leaf curl alphasatellite (PaLCuA) (Table 1). Similar to begomoviruses, with BLASTn analysis, GDarSLA sequences were found to be homologous (95%–99%) to recent alphasatellites isolated primarily from CCRI-Multan (EU384607, EU384622, EU384653, etc.), Vehari region of Pakistan (KX656836-KX656840, KX656852), and India (MF929032, MF929023). Similarly, GDavSLA sequences found in this study were 94%–99% identical to sequences reported from *G. davidsonii* (EU384652, EU384653) and *G. hirsutum* (KX656847-KX656848) from Pakistan. Other alphasatellites species identified in this study showed high sequence identity (93%–100%) to PaLCuA, AYSVA and CLCuBuA sequences isolated from the Indian subcontinent. BLASTn results demonstrated that amino acid alignment of alphasatellite Rep protein described two variants; a Rep protein with 315 amino acids and another Rep like protein with 295 amino acids (S1 File). This kind of natural alphasatellite Rep variant has also been identified previously [31] and occurs due to frame shift mutations that truncate the Rep protein C terminally. 

Two species of betasatellites were detected from *G. thurberi*. BLASTn analysis indicated the closest identity (95%–98%) with cotton leaf curl Multan betasatellite (CLCuMuB)-Shahdadpur strain (accession no. KX697600, KX697602) and -Burewala strain (accession no. KX697599) isolated from Vehari-Punjab-Pakistan [26]. BLASTn analysis of okra leaf curl betasatellite (OLCuB) showed their best match (92%–96% sequence identity) with OLCuB sequences reported from India and Pakistan (accession no. AJ316029, KJ437507). The predicted βC1 ORF was of typical size of 118 amino acids for *begomovirus*-associated betasatellites (Table S3).

### 2.3. Begomovirus/Satellites are Phylogenetically Related to the Recent Cotton Leaf Curl Disease Complex 

**Figure 2:**
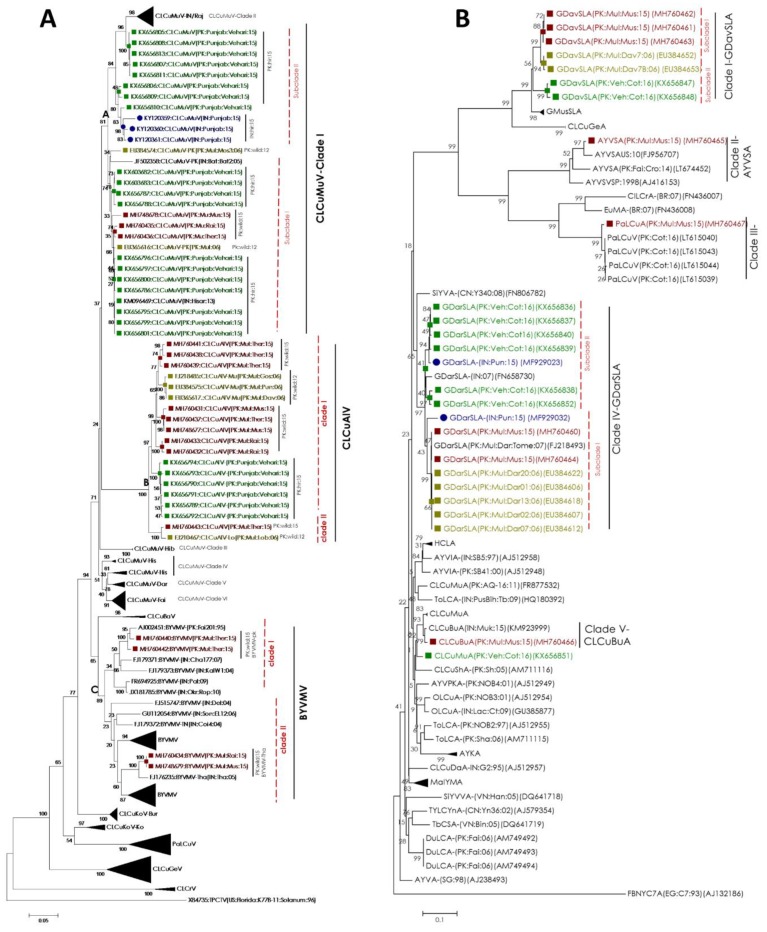
Phylogeny of monopartite and bipartite *begomovirus* DNA components A and alphasatellites. Neighbor-joining (NJ) phylogenetic dendrogram of monopartite begomoviruses and DNA-A component of bipartite begomoviruses (**A**) and alphasatellites (**B**), generated in this study from non-cultivated cotton species (group named as “PK:wild:15”) with related sequences from database. All DNA-A and alphasatellite sequences found in the current study (Multan, Pakistan, 2015) are represented by solid maroon squares and colored maroon. Sequences reported from non-cultivated cotton species from Multan-Pakistan (2012) [31] are represented by solid olive square and are colored olive (group named as Pk:wild:12). Sequences isolated from cultivated cotton from Vehari-Pakistan (2015) [26] and Punjab-India (2015) [27] are shown by solid green squares and solid blue rounds, respectively. CLCuMuV clades in panel A are illustrated according to recent paper [33]. *Begomovirus* and alphasatellite species names have been abbreviated according to the revised nomenclature [32].

A phylogenetic analysis was performed on the full-length sequences of DNA-A (and monopartite begomoviruses), DNA-B, alphasatellites and betasatellites with their respective similar isolates available in the database, based on MUSCLE alignment. A neighbor-joining (NJ) phylogenetic tree of DNA-A, reconstructed with DNA-A sequences in this study and other related *begomovirus* isolates, resulted in separation into three major groups (A–C; Figure 2a). Group A consisted of CLCuMuV sequences, which was further divided into different clades according to different strains of CLCuMuV (clade I–clade VI) as described earlier [33]. The first CLCuMuV clade bifurcated into two subclades. Corroborating with our BLASTn results, CLCuMuV sequences isolated in this study were found to be phylogenetically affiliated with recently reported CLCuMuV sequences from *G. hirsutum* (Vehari-Punjab-Pakistan) [26] (Figure 2). A phylogenetic relationship with CLCuMuV sequences isolated from non-cultivated cotton species, *G. lobatum*, *G. punctatum* and *G. gossypoides* in 2012 [31] confirmed their consistent presence in that herbarium (subclade I). In line with the phylogenetic relationship described earlier by Datta et al., sub-clade II was comprised of the CLCuMuV sequences isolated from *G. hirsutum* during the recent outbreaks in India and Pakistan that confirmed the authenticity of our phylogenetic analysis. Taken together, CLCuMuV isolates harbored by non-cultivated cotton species since 2000s, are the possible progenitor CLCuMuV isolates now prevailing in recent outbreaks in cultivated cotton fields.

Group B in the phylogenetic tree of DNA-A bifurcated into two clades according to different strains of CLCuAlV. Clade I represents the close relationship among CLCuAlV-Mul isolates reported here, and isolates reported from non-cultivated cotton species in 2012 by Nawaz-ul-Rehman et al. and cultivated cotton species in 2015 by Zubair et al. from Pakistan. Clade II consisted on CLCuAlV-Lo isolates reported here and sequences reported from other non-cultivated cotton species in 2012, thus providing an insight into the possible origin of CLCuAlV reemergence.

Group C in the phylogenetic tree of DNA-A consisted of BYVMV isolates that sub-grouped further into two clades (Figure 2a). Sequences in the first clade consisted of sequences isolated from *G. thurberi* that rooted with BYVMV-Pakistan strain (accession no. AJ002451) reported from Pakistan. Sequences in the clade II consisted of sequences isolated from *G. mustelinum* and *G. raimondii*, rooted with Thanagan strain of BYVMV (accession no. FJ176235) isolated from India. Detection of BYMV species from non-cultivated cotton species in this study is distinct from all the previous key reference studies [26,27,31], suggesting its recent introduction in non-cultivated cotton species being maintained in Multan-Pakistan.

A phylogenetic dendrogram of alphasatellite was constructed using isolates obtained here and other representative sequences of different alphasatellite species reported from Pakistan and India [26,27]. The phylogenetic tree segregated into five clades (Figure 2b). Phylogenetic affiliation of alphasatellites species obtained here and recently reported reference sequences is indicated in clade 1 and V that was also in accordance with the BLASTn results. Sequences in the first clade rooted with GDavSLA (clade I-subclade I), which were originally isolated from the non-cultivated cotton species, *G. davidsonii* in 2012, and therefore named as GDavSLA [31]. Alphasatellites in clade V segregated with GDarSLA reported from *G. darwinii* (subclade I) and *G. hirsutum* (subclade II) from Pakistan [26,31]. Similar to GDavSLA, GDarSLA isolates were also closely related to the sequences reported from non-cultivated cotton species. Consistent with the previous findings [27], GDarSLA, recently reported in *G. hirsutum* from Pakistan and India, co-segregated indicating their close similarity (clade IV, subclade II). The other three alphasatellite isolates segregated with CLCuBuA, PaLCuA and AYVSA, previously reported from croton and cotton (Figure 2b, clade II–IV). 

**Figure 3:**
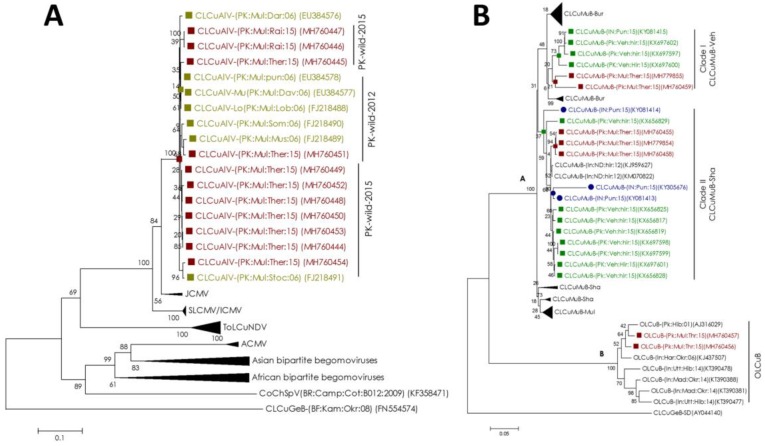
Phylogeny of DNA-B component of bipartite begomoviruses and betasatellites. (**A**) Neighbor-joining (NJ) phylogenetic dendrogram of cotton leaf curl Alabad virus (CLCuAlV) DNA-B reported in this study (group named as “PK:wild:2015”) and other related bipartite begomoviruses. Other representative sequences (from Asia, Africa and South America) have been included in the phylogenetic analysis but has been compressed in the final figure for better representation. (**B**) Phylogenetic dendrogram of betasatellites reconstructed with cotton leaf curl Multan betasatellite (CLCuMuB) and okra leaf curl betasatellite (OLCuB) found in the current study with other related sequences. Some CLCuMuB branches representing different strains (that are irrelevant to the scope of current study) have been collapsed to avoid complexity. All the sequences reported in this study are colored maroon with solid maroon squares and closely related betasatellite sequences isolated from Pakistan and India (2015) from cultivated cotton (*G. hirsutum*) are colored green and blue respectively. Sequences reported from the pioneer study on non-cultivated cotton species [31] have been represented by solid olive squares and olive text (group named as PK:wild:2012). *Begomovirus* and betasatellite species names have been abbreviated according to the standard nomenclature [32] and CLCuMuB strains have been assigned names as described earlier [10]. Both dendrograms are supported by 1000 bootstrap value and were arbitrarily rooted with cotton leaf curl gezira leaf curl betasatellite (CLCuGeB) as an outgroup.

Consistent with BLASTn analysis, phylogeny of DNA-B represented the segregation with CLCuAlV DNA-B component previously reported from *G. punctatum*, *G. darwinii*, *G. mustelinum*, *G. stocksii* and *G. somalense* cotton species [31] (Figure 3a). Segregation of CLCuAlV DNA-B with the DNA-B component of ICMV, SLCMV and JCMV indicate that it probably originated from one of these or closely related viruses (Figure 3a). 

The phylogenetic dendrogram of betasatellites segregated into two groups (Figure 3b); the first group (A) consisted of CLCuMuB that further separated into two clades; and the second group (B) comprised of betasatellites isolated from *G. thurberi* that segregated with Indian isolates of OLCuB. Among the two clades within the group A, sequences in the first clade were closely related to CLCuMuB-Vehari strain reported from *G. hirsutum* in 2017 from Pakistan (KX697600, KX697597 and KX697602) and India (KY018415) [26,27]. The other clade contained betasatellite sequences rooted with Shadadpur strain of CLCuMuB (recombinant strain of CLCuMuB recently proposed by Zubair et al.) [10], isolated from *G. hirsutum* from Pakistan (KX697599, KX697598, KX656825, etc.) and India (KY305676, KY081413 and KY081414) [26,27], which further refined our hypothesis that the spread of *begomovirus* (CLCuMuV) and betasatellite (CLCuMuB) may have occurred from non-cultivated to cultivated cotton species. The other clades in the phylogenetic tree represent other strains of CLCuMuB prevailing in the Indian subcontinent.

### 2.4. Begomovirus Levels are Reduced in the Presence of an Alphasatellite 

**Figure 4:**
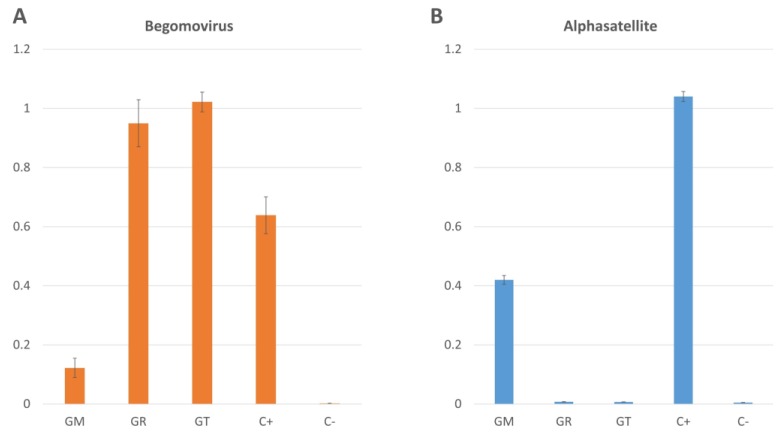
Quantitative analysis of *begomovirus* and alphasatellite. (**A**) qPCR with *begomovirus* specific primers where GM, GR and GT represent *G. mustelinum*, *G. raimondii* and *G. thurberi*, respectively. Cotton leaf curl disease (CLCuD) infected *G. hirsutum* plants were taken as positive control C+ and CLCuD free *G. hirsutum* was taken as negative control C-. (**B**) qPCR with alphasatellite specific primers. The titer of each component is given in ng/μg of genomic DNA on the y-axis and is the mean of three replications. The error bars are the divergence from mean quantified value.

To confirm the presence of alphasatellites in tetraploid cotton species, their absence in diploid species, and to identify their levels in comparison with *begomovirus*, a quantitative analysis was performed using qPCR. Analysis indicated that the amount of *begomovirus* in *G. raimondii* and *G. thurberi* was one and a half times as much as that in the CLCuD infected cultivated cotton *G. hirsutum*; whereas in *G. mustelinum*, virus titer was five times less than that in *G. hirsutum* (Figure 4: panel A). However, comparative analysis of alphasatellite levels in the same samples indicated that the titer in *G. mustelinum* was about two times less than that in *G. hirsutum* and no signal was detected in *G. raimondii* and *G. thurberi* (Figure 4: panel B). This negative correlation was confirmed by negative correlation coefficient on a scatter plot (Figure S4). Overall, qPCR analysis indicated a negative correlation among *begomovirus* and alphasatellite levels, which is consistent with our previous observations in *G. hirsutum* [34]. Surprisingly, in the case of the betasatellite, the levels were below the detection limit of quantitative thermal cycler, which indicated that although betasatellites are present, their level is significantly lower compared to alphasatellites and begomoviruses.

## 3. Discussion

Cultivated cotton *G. hirsutum* is highly susceptible to CLCuD in the Indian subcontinent, while many non-cultivated cotton species show natural immunity against CLCuD [35,36]. The first epidemic of CLCuD (during the 1990s) was associated with multiple begomoviruses, i.e., CLCuAlV, CLCuMuV, CLCuKoV, ToLCBaV, PaLCuV and CLCuRaV [21]. However, during the second epidemic (in the 2000s), a single recombinant strain of *begomovirus* (CLCuKoV-Bur) and betasatellite (CLCuMuB^Bur^) were identified as dominant causal agents [37]. Recently we have identified that a bipartite *begomovirus* tomato leaf curl New Delhi virus (ToLCNDV) is spreading widely in the cotton growing areas of Punjab and Sindh provinces in Pakistan [38,39]. This was followed by the reports of reemergence of the first epidemic’s CLCuD complex (CLCuAlV, CLCuMuV and CLCuRaV) in the cultivated cotton in Pakistan [26] and India [27]. The actual reason for this reemergence is as yet unknown and can only be speculated [40], but the data combined with previous observations [37], suggests that rapid evolution of geminiviruses and may lead to a future CLCuD epidemic in the Old World species [28].

We hypothesized that, since non-cultivated cotton species have been reported to maintain viruses from the first epidemic [31], they may have a role in the reemergence of CLCuD-associated begomoviruses. Therefore, we sequenced and characterized begomoviruses and associated satellites from the non-cultivated cotton species. Phylogenetic analysis indicated that the begomoviruses (CLCuMuV and CLCuAlV), betasatellites (CLCuMuB) and alphasatellites (GDarSLA and GDavSLA) isolated in this study are closely related to, and share maximum sequence identity with the begomoviruses and alpha/betasatellites from the studies reporting the recent reemergence of the first epidemic’s CLCuD complex [26,27] (Figure 2 and Figure 3). This indicated the retention, and possible movement of CLCuD begomoviruses and associated satellites from non-cultivated cotton species to cultivated cotton. Consistent with the previous studies, we did not find the recombinant strain of CLCuKoV-Bur, the dominant responsible causal agent of the second epidemic of CLCuD in the Indian subcontinent and signified the epidemiology shift of CLCuD. The possible reason for the absence of a virulent strain CLCuKoV-Bur can only be speculated. To act as a good reservoir for virus, the alternate host species must maintain viruses that produce little to no symptoms on that reservoir plant. This allows the alternate host plant to survive and transmit virus over generations. A second reason could be the complete resistance of these wild species to the viruses from the second epidemic. Alternate virus-host species can maintain less virulent viruses but can have a complete resistance to more virulent species. The viruses from the second epidemic, CLCuKoV-Bur for example, are considered to be the most virulent species among all CLCuVs [37], and could explain its absence in wild cotton species.

The other significant aspect of our study is identification of previously unidentified begomoviruses and satellite molecules from non-cultivated cotton species. We performed a comparative analysis of begomoviruses and satellites in the present study with the previous study performed on non-cultivated cotton species [31]. The methodology used previously was based on cloning and Sanger sequencing. Here we used RCA followed by NGS to identify begomoviruses and associated satellites. We studied *G. raimondii* which was not previously evaluated for the presence of begomoviruses. Begomoviruses or satellites were not detected in *G. thurberi* in the 2012 study [31], while in this study, three *begomovirus* species (CLCuMuV-Pk, CLCuAlV-Mu and BYVMV-Th) were detected in *G. raimondii.* In *G. thurberi*, three *begomovirus* species with four distinct strains (CLCuMuV-Pk, CLCuAlV-Mu, CLCuAlV-Lob and BYVMV-Pk) were identified in association with two betasatellites species, CLCuMuB and OLCuB (Table 1) with the complete absence of any alphasatellite species. In *G. mustelinum,* three *begomovirus* species CLCuMuV-Pk, CLCuAlV-Mu and BYVMV-Th were detected in association with five alphasatellites including GDarSLA, GDavSLA, AYVSA, CLCuBuA and PaLCuA (Table 1) and no betasatellite was detected. *Begomovirus* diversity in *G. mustelinum* was more variable compared to the previous study. In 2012, CLCuMuV and CLCuAlV were not detected in *G. mustelinum*, on the other hand, CLCuKoV was identified [31]. While in the present study, the opposite was true and we identified CLCuMuV and CLCuAlV from *G. mustelinum* but we did not detect CLCuKoV. Another interesting aspect in this study was the complete absence of betasatellites in *G. mustelinum*, while in the previous study, a mutant version of betasatellite lacking aβC1 gene was detected [31]. This might correlate with the current study in the sense that a betasatellite lacking its important βC1 gene may have no or very little function in the disease development in its host and was completely removed from the disease complex over time. Comparing the alphasatellite diversity in this species, GDarSLA and GMusSLA were identified in 2012, but in the current study, GMusSLA was replaced by GDavSLA. Our data represents the important changes that occurred in the disease complex over time since 2012. Overall, our data provided valuable new information, in addition to confirming previous reports [31]. 

Surprisingly, we detected alphasatellites only in tetraploid cotton species *G. mustelinum,* and not in the diploid cotton species *G. raimondii* and *G. thurberi.* Likewise, we found betasatellites only in *G. thurberi,* and not in the other cotton species under study. However, only a mutant version of betasatellite lacking its βC1 gene was identified in *G. mustelinum* in the previous study [31]. Given the limited number of species studied here, the pattern in host-specific selection of alphasatellites and betasatellites is only speculative. Further research on other non-cultivated species and on a wider scale will provide a deeper insight. Comparative analysis of alphasatellite and *begomovirus* levels indicated that virus levels in tetraploid cotton were significantly lower where alphasatellites were present. Alternatively, virus levels were higher in diploid cotton species where alphasatellites were absent. Previous studies have shown that alphasatellites suppress the symptoms of *begomovirus* disease and decrease the virus titer in infected plant cells [41]. We found two alphasatellite variants, one with a complete Rep (315 amino acids) that is the most commonly found alphasatellite and the other with a truncated Rep (295 amino acids) previously reported in non-cultivated cotton from the same Multan collection [31]. This kind of natural variant is a result of frame shift mutations and might have some role in avoiding detection by the host defense mechanism [5]. Consistent with previous findings [34], our results support the relationship between the occurrence of alphasatellites and the reduction of virus level in the presence of alphasatellites under field conditions, and suggest a potential role for alphasatellites in CLCuD. Overall, the differential detection of the satellites in the present study is interesting. Further studies should explain a possible mechanism for this observation.

In conclusion, the detection of the first CLCuD epidemic-associated begomoviruses/satellites in the non-cultivated cotton species, and their close phylogenetic affiliation with the recent CLCuD complex (in Pakistan and India) is quite surprising. Since virus and satellite molecules from both recent studies also have phylogenetic relatedness among them, there must be a common region from where all these virus and satellite species are being harbored and spread to the nearby cotton fields whenever the environmental conditions are favorable. This study identified a common origin and signified the idea that non-cultivated cotton species may act as reservoirs for CLCuD-associated begomoviruses/satellites and could play a role in the reemergence of CLCuMuV, CLCuAlV and CLCuKoV in cotton fields of Pakistan and India in 2015. Apart from the strong phylogenetic relationship among begomoviruses and satellites reported here and recently from Punjab-Pakistan/India, they also have high sequence identity with the isolates previously reported from non-cultivated cotton species in 2012 by Nawaz-ul-Rehman et al. [31]. This suggests that these virus/satellite species are being maintained by non-cultivated cotton species over a long period of time and possibly spread to the nearby regions when suitable environment prevails. Moreover, the levels of alphasatellites are negatively correlated with the levels of begomoviruses and differential detection of alpha/betasatellites depending on the genome of the *Gossypium* species opens up a new area of research. Future studies will provide a deeper insight into the species-specific infection of alphasatellites and betasatellites.

## 4. Materials and Methods 

### 4.1. Sample Collection, DNA Extraction and Rolling Circle Amplification

For the molecular detection of possible virus/satellite, leaf samples from *G. mustelinum* (AD4), *G. raimondii* (D5) and *G. thurberi* (D1), maintained at the Central Cotton Research Institute (CCRI) Multan (Punjab, Pakistan, 30.1°N, 71.4°E), were collected in 2015 (Figure 1). Leaf samples were collected from three independent biological replicates. For comparison, leaf samples were also collected from asymptomatic cultivated cotton, *G. hirsutum*. In total, ten samples were processed for DNA extraction, RCA and next generation sequencing (NGS). Total DNA was extracted from 100 mg of leaf tissues using the CTAB method [42]. RCA was performed to amplify all circular molecules in the DNA extracts according to protocol described earlier [43]. RCA enriched product was purified using ethanol precipitation and was proceeded for NGS. 

### 4.2. Library Preparation, Sequencing, Assembly and Analysis

The details, from library preparation to the data analysis, have been described elsewhere [26]. “The Illumina NeoPrep automation system (Illumina, San Diego, CA) was used with library kit, Illumina #NP-101-1001, ‘TruSeq Nano DNA Library Kit for NeoPrep’, which includes the adapter set “TruSeq LT”. The target insert size was 350 bp, with size selection performed by the NeoPrep instrument. The actual lower size limit of the libraries was ~300 bp as measured by the Agilent 2200 TapeStation (Santa Clara, CA). Sequencing was performed on the Illumina MiSeq, v2 chemistry, 2 × 150 bp. The MiSeq Reporter software was set to automatically trim the adaptors. These sequences were processed using CLC Genomics Work Bench 7.5 [44]. The paired-end reads obtained from the Illumina MiSeq Sequencer pipeline were subjected to quality filtering using a quality score of 0.001 and a Phred quality score of 30. De novo and reference-guided assemblies were made. Reference-guided assembly of *begomovirus* was made using a comprehensive list of begomoviruses prepared based on the information provided at the International Committee on Taxonomy of Viruses (ICTV) website (www.ictvonline.org). For reference-guided assembly of alphasatellite and betasatellite, sequences present in GenBank were used. All sequences were searched for similarity against the NCBI non-redundant nucleotides database (nt) using the BLAST + tool, provided by NCBI” [26].

### 4.3. Alignment, SDT and Phylogeny

For *begomovirus* DNA-A isolates, a neighbor joining phylogenetic tree was inferred from aligned sequences of begomoviruses (CLCuMuV, CLCuAlV and BYVMV) isolated in this study together with 235 other sequences from seven major cotton infecting monopartite *begomovirus* species with their representative strains reported so far in OW and New World (NW). For alphasatellites, neighbor joining dendrogram was constructed using 27 representative alphasatellite species reported from OW and NW along with alphasatellite sequences reported here. Neighbor joining phylogenetic tree for DNA-B isolates was inferred using aligned sequences isolated here together with other DNA-B sequences of 19 representative bipartite *begomovirus* species reported from Asia, Africa and Brazil. Similarly, betasatellite phylogenetic dendrogram was inferred using aligned betasatellite sequences isolated here along with the representative strains of CLCuMuB and OLCuB reported so far. 

Sequence fasta files were imported in MEGA6 for alignment and phylogenetic analysis. All the sequences were aligned using MUSCLE option in MEGA6 [45]. Same alignments were used for the construction of neighbor joining phylogenetic trees in MEGA6 [45]. All phylogenetic trees were supported with 1000 bootstrap values to validate the phylogenetic analysis and were further edited to adapt to the recent nomenclature. For *begomovirus* species and strain demarcation, sequence demarcation tool (SDT) v1.2 was used to perform MUSCLE alignment and construct identity matrices following the methodology explained earlier [46].

### 4.4. Recombination Analysis

For the detection of any recombination events, recombination analysis was performed using virus sequences isolated in this study along with representative begomoviruses sequences used earlier [26] with the Recombination Detection Program RDP4 beta v 4.96 using a set of algorithms RDP, MAXCHI, CHIMAERA, 3SEQ, GENECONV, SISCAN, BOOTSCAN and LARD [47]. Sequences were aligned using MUSCLE alignment in MEGA6 [45] and exported fasta files were used as input for RDP4. Recombination analysis was performed using default setting with cutoff *p* value 0.05.

### 4.5. Quantification with qPCR

Genomic DNA of non-cultivated cotton species was used in qPCR experiment, where CLCuD-infected and CLCuD-free *G. hirsutum* DNA samples were used as positive and negative controls, respectively. We have provided a detailed qPCR methodology, adapted specifically for the detection of CLCuD complex in cotton, elsewhere [34]. “The primers used in the qPCR analyses were DNA_A_qPCR_Forward (CCTTTAATCATGACTGGCTT)/DNA_A_qPCR_Reverse (CATTTCCATCCGAACATTC) for the *begomovirus* genome or DNA-A component, DNA_B_qPCR_Forward (GCCCATGATTCGTTCGGAC)/DNA_B_qPCR_Reverse (CACGTGGTACTGGAATATCGCA) for DNA-B and Betasatellite_qPCR_Forward (GATTTGACTTATATTGGGCCAATTTAAT)/Betasatellite_qPCR_Reverse (GATACTATCCACAAAGTCACCATCGCTAAT) for betasatellites [39]. qPCR reactions consisted of a total volume of 25 μL with 12.5 μL of SYBR Green Super Mix (Thermo Fisher Scientific, Waltham, MA USA), 0.25 μL of each primer (0.1 μM each), 2.5 μL of DNA (25 ng) and 9.5 μL water. The cycling conditions were an initial 94 °C for 10 min, followed by 40 cycles of 30 s at 94 °C, 30 s at 57 °C, 30 s at 72 °C, followed by melt curve analyses. Reactions were performed in a 96 well microtiter plate format using an iQ5 thermal cycler (Bio-Rad, Hercules, CA USA). The 18S ribosomal RNA gene was used as a reference gene to normalize DNA levels in samples. Each sample was run in triplicate” [34].

## Figures and Tables

**Table 1:** *Begomovirus*, alphasatellite and betasatellite species isolated from *G. mustelinum*, *G. raimondii* and *G. thurberi*.

Species	*Begomovirus*/alphasatellite/betasatellite	Genomic Component	Strain
***G. mustelinum* (AADD)**	Cotton leaf curl Multan virus	Monopartite	Pakistan
	Bhendi yellow vein mosaic virus	Monopartite	Thanagan
	Cotton leaf curl Alabad virus	DNA-A	Multan
	Cotton leaf curl Alabad virus	DNA-B	Multan
	Cotton leaf curl Burewala alphasatellite	Alphasatellite	N. A
	Ageratum yellow vein Singapore alphasatellite	Alphasatellite	N. A
	*Gossypium darwinii* symptomless alphasatellite	Alphasatellite	N. A
	*Gossypium davidsonii* symptomless alphasatellite	Alphasatellite	N. A
	Papaya leaf curl alphasatellite	Alphasatellite	N. A
***G. raimondii* (DD)**	Bhendi yellow vein mosaic virus	Monopartite	Thanagan
	Cotton leaf curl Alabad virus	DNA-A	Multan
	Cotton leaf curl Alabad virus	DNA-B	Multan
	Cotton leaf curl Multan virus	Monopartite	Pakistan
***G. thurberi* (DD)**	Bhendi yellow vein mosaic virus	Monopartite	Pakistan
	Cotton leaf curl Alabad virus	DNA-A	Multan
	Cotton leaf curl Alabad virus	DNA-B	Multan
	Cotton leaf curl Alabad virus	DNA-A	Lobatum
	Cotton leaf curl Alabad virus	DNA-B	Lobatum
	Cotton leaf curl Multan virus	Monopartite	Pakistan
	Cotton leaf curl Multan betasatellite	Betasatellite	Shahdadpur
	Cotton leaf curl Multan betasatellite	Betasatellite	Burewala
	Okra leaf curl betasatellite	Betasatellite	N. A

## Supplementary Materials

The following are available online at https://www.mdpi.com/2223-7747/8/5/127/s1, **Figure S1.** SDT analysis for cotton leaf curl Multan virus (CLCuMuV), **Figure S2.** SDT analysis for cotton leaf curl Alabad virus (CLCuAlV), **Figure S3.** SDT analysis for bhendi yellow vein mosaic virus (BYVMV), **Figure S4.** Negative correlation between *begomovirus* and alphasatellite levels. The scatter plot in based on the titers of *begomovirus* and alphasatellite in the non-cultivated cotton species used in this study and measured using qPCR, **S1 File.** Amino acid alignment for alphasatellite Rep protein of *Gossypium darwinii* symptomless alphasatellite (GDarSLA), *Gossypium davidsonii* symptomless alphasatellite (GDavSLA) and cotton leaf curl Burewala alphasatellite (CLCuBuA), **Table S1.** Open reading frame (ORF) analysis of DNA-A components of monopartite and bipartite begomoviruses, **Table S2.** Open reading frame (ORF) analysis of DNA-B components of bipartite begomoviruses, **Table S3.** Open reading frame (ORF) analysis of begomoviruses associated betasatellites and alphasatellites.
