# Supplementary material for: Non-cultivated Cotton Species (Gossypium spp.) Act as a Reservoir for Cotton Leaf Curl Begomoviruses and Associated Satellites
Source: Plants (Basel). 2019 May 14;8(5):127. doi: 10.3390/plants8050127 (PMC6571856; doi:10.3390/plants8050127)
Supplement: Supplementary file 1 [file plants-08-00127-s001.zip › Supplementary Materials/Table S1.docx]

| **Sample** | **Host** | **Clone name** | **Virus component** | **Location (Province-District)** | **Accession no.** | **Size (nt)** | **Position (nucleotide coordinates/coding capacity)** | | | | | | |
| --- | --- | --- | --- | --- | --- | --- | --- | --- | --- | --- | --- | --- | --- |
|  |  |  |  |  |  |  | **Rep** | **TrAP** | **REn** | **C4** | **C5** | **CP** | **V2** |
| MW19 | *G. mustelinum* | SSR1 | CLCuAlV | Punjab-Multan | MH748677 | 2733 | 1500-2591/363 | 1199-1603/134 | 1054-1458/134 | 2177-2434/85 | 695-946/83 | 281-1051/256 | 121-486/121 |
|  | *G. mustelinum* | SSR5 | CLCuMuV | Punjab-Multan | MH748678 | 2739 | 1496-2584/362 | 1195-1599/134 | 1050-1454/134 | 2128-2430/100 | 625-942/105 | 277-1047/256 | 117-482/121 |
|  | *G. mustelinum* | SSR29 | BYVMV | Punjab-Multan | MH748679 | 2742 | 1499-2587/362 | 1150-1602/150 | 1053-1457/134 | 2131-2439/102 | 628-984/118 | 280-1050/256 | 120-485/121 |
| MW20 | *G. raimondii* | SSR7 | CLCuAlV | Punjab-Multan | MH760432 | 2728 | 1495-2586/363 | 1146-1598/150 | 1049-1453/134 | 2172-2429/85 | 624-941/105 | 276-1046/256 | 116-481/121 |
|  | *G. raimondii* | SSR10 | CLCuAlV | Punjab-Multan | MH760433 | 2734 | 1501-2592/363 | 1152-1604/150 | 1055-1459/134 | 2178-2435/85 | 630-947/105 | 282-1052/256 | 122-487/121 |
|  | *G. raimondii* | SSR11 | BYVMV | Punjab-Multan | MH760434 | 2742 | 1499-2587/362 | 1150-1602/150 | 1053-1457/134 | 2131-2439/102 | 628-984/118 | 280-1050/256 | 120-485/121 |
|  | *G. raimondii* | SSR12 | CLCuMuV | Punjab-Multan | MH760435 | 2738 | 1495-2583/362 | 1146-1598/150 | 1049-1453/134 | 2127-2429/100 | 624-941/105 | 276-1046/256 | 116-481/121 |
| MW23 | *G. thurberi* | SSR3 | CLCuAlV | Punjab-Multan | MH760431 | 2729 | 1496-2587/363 | 1195-1599/134 | 1050-1454/134 | 2173-2430/85 | 691-942/83 | 277-1047/256 | 117-482/121 |
|  | *G. thurberi* | SSR13 | CLCuMuV | Punjab-Multan | MH760436 | 2737 | 1495-2583/362 | 1146-1598/150 | 1049-1453/134 | 2127-2429/100 | 624-941/105 | 276-1046/256 | 116-481/121 |
|  | *G. thurberi* | SSR15 | CLCuAlV | Punjab-Multan | MH760437 | 2733 | 1500-2591/363 | 1199-1603/134 | 1054-1458/134 | 2177-2434/85 | 695-946/83 | 281-1051/256 | 121-486/121 |
|  | *G. thurberi* | SSR16 | CLCuAlV | Punjab-Multan | MH760438 | 2658 | 1502-2593/363 | 1153-1605/150 | 1056-1502/148 | 2179-2436/85 | 631-948/105 | 283-1053/256 | 123-488/121 |
|  | *G. thurberi* | SSR17A | CLCuAlV | Punjab-Multan | MH760439 | 2728 | 1495-2586/363 | 1146-1598/150 | 1049-1453/134 | 2172-2429/85 | 624-941/105 | 276-1046/256 | 116-481/121 |
|  | *G. thurberi* | SSR19 | BYVMV | Punjab-Multan | MH760440 | 2738 | 1499-2590/363 | 1150-1602/150 | 1053-1457/134 | 2131-2439/102 | 628-984/118 | 280-1050/256 | 120-485/121 |
|  | *G. thurberi* | SSR25 | CLCuAlV | Punjab-Multan | MH760441 | 2734 | 1500-2591/363 | 1151-1603/150 | 1054-1458/134 | 2177-2434/85 | 629-946/105 | 281-1051/256 | 121-486/121 |
|  | *G. thurberi* | SSR26 | BYVMV | Punjab-Multan | MH760442 | 2739 | 1500-2591/363 | 1151-1603/150 | 1054-1458/134 | 2132-2440/102 | 629-985/118 | 281-1051/256 | 121-486/121 |
|  | *G. thurberi* | SSR27 | CLCuAlV | Punjab-Multan | MH760443 | 2730 | 1500-2588/362 | 1151-1603/150 | 1054-1458/134 | 2141-2431/96 | 629-946/105 | 281-1051/256 | 121-483/120 |

**Table S1:** ORF analysis for monopartite begomovirus or DNA-A component of bipartite begomoviruses found in *G. mustelinum, G. raimondii*, *G. thurberi*. Nucleotide coordinates and coding capacity (amino acids) for each gene is given.
