# Supplementary material for: Non-cultivated Cotton Species (Gossypium spp.) Act as a Reservoir for Cotton Leaf Curl Begomoviruses and Associated Satellites
Source: Plants (Basel). 2019 May 14;8(5):127. doi: 10.3390/plants8050127 (PMC6571856; doi:10.3390/plants8050127)
Supplement: Supplementary file 1 [file plants-08-00127-s001.zip › Supplementary Materials/Table S2.docx]

**Table S2:** Open reading frame (ORF) analysis of DNA-B components of bipartite begomoviruses. Nucleotide coordinates and coding capacity (amino acids) for each gene is given.

| **Sample** | **Host** | **Clone name** | **Virus component** | **Location (Province-District)** | **Accession no.** | **Size (nt)** | **Position (nucleotide coordinates/no. of amino acids)** | |
| --- | --- | --- | --- | --- | --- | --- | --- | --- |
|  |  |  |  |  |  |  | **MP** | **NSP** |
| MW19 | *G. mustelinum* | SSR2 | CLCuAlV | Punjab-Multan | MH760444 | 2730 | 1343-2167/274 | 516-1334/272 |
|  | *G. mustelinum* | SSR4 | CLCuAlV | Punjab-Multan | MH760445 | 2672 | 1377-2201/274 | 550-1368/272 |
| MW20 | *G. raimondii* | SSR8 | CLCuAlV | Punjab-Multan | MH760446 | 2680 | 1377-2201/274 | 550-1368/272 |
|  | *G. raimondii* | SSR9 | CLCuAlV | Punjab-Multan | MH760447 | 2734 | 1344-2168/274 | 517-1335/272 |
| MW23 | *G. thurberi* | SSR14 | CLCuAlV | Punjab-Multan | MH760448 | 2732 | 1342-2166/274 | 515-1333/272 |
|  | *G. thurberi* | SSR17 | CLCuAlV | Punjab-Multan | MH760449 | 2678 | 1376-2200/274 | 543-1367/274 |
|  | *G. thurberi* | SSR18 | CLCuAlV | Punjab-Multan | MH760450 | 2671 | 1376-2200/274 | 549-1367/272 |
|  | *G. thurberi* | SSR20 | CLCuAlV | Punjab-Multan | MH760451 | 2676 | 1374-2198/274 | 550-1365/271 |
|  | *G. thurberi* | SSR21 | CLCuAlV | Punjab-Multan | MH760452 | 2777 | 1369-2199/276 | 542-1360/272 |
|  | *G. thurberi* | SSR22 | CLCuAlV | Punjab-Multan | MH760453 | 2705 | 1378-2202/274 | 551-1369/272 |
|  | *G. thurberi* | SSR28 | CLCuAlV | Punjab-Multan | MH760454 | 2755 | 1304-2128/274 | 522-1295/257 |
