# Supplementary material for: Non-cultivated Cotton Species (Gossypium spp.) Act as a Reservoir for Cotton Leaf Curl Begomoviruses and Associated Satellites
Source: Plants (Basel). 2019 May 14;8(5):127. doi: 10.3390/plants8050127 (PMC6571856; doi:10.3390/plants8050127)
Supplement: Supplementary file 1 [file plants-08-00127-s001.zip › Supplementary Materials/Table S3.docx]

**Table S3:** Open reading frame (ORF) analysis of begomoviruses associated betasatellites and alphasatellites. Nucleotide coordinates and coding capacity (amino acids) for each gene is given.

|  | **Betasatellites found in this study through NGS** | | | | | | | |
| --- | --- | --- | --- | --- | --- | --- | --- | --- |
| **Sample** | **Host** | **Clone name** | **Virus component** | **Location (Province-District)** | **Accession no.** | **Size (nt)** | **Position (nucleotide coordinates/no. of amino acids)** | |
|  |  |  |  |  |  |  | **BC1** | **Rep** |
| MW23 | *G. thurberi* | SSR30 | CLCuMuB | Punjab-Multan | MH760455 | 1371 | 195-551/118 | _ |
|  | *G. thurberi* | SSR31 | OLCuB | Punjab-Multan | MH760456 | 1358 | 192-548/118 | _ |
|  | *G. thurberi* | SSR32 | OLCuB | Punjab-Multan | MH760457 | 1357 | 192-548/118 | _ |
|  | *G. thurberi* | SSR33 | CLCuMuB | Punjab-Multan | MH760458 | 1369 | 195-551/118 | _ |
|  | *G. thurberi* | SSR34 | CLCuMuB | Punjab-Multan | MH779854 | 1372 | 194-550/118 |  |
|  | *G. thurberi* | SSR35 | CLCuMuB | Punjab-Multan | MH779855 | 1372 | 411-551/46 |  |
|  | *G. thurberi* | SSR36 | CLCuMuB | Punjab-Multan | MH760459 | 1366 | 195-551/118 | _ |
|  | **Alphasatellites found in this study through NGS** | | | | | | | |
| MW19 | *G. mustelinum* | SSR40 | GDarSLA | Punjab-Multan | MH760460 | 1377 | _ | 70-1017/315 |
|  | *G. mustelinum* | SSR42 | GDavSLA | Punjab-Multan | MH760461 | 1216 | _ | 58-945/295 |
|  | *G. mustelinum* | SSR41 | GDavSLA | Punjab-Multan | MH760462 | 1216 | _ | 58-945/295 |
|  | *G. mustelinum* | SSR43 | GDavSLA | Punjab-Multan | MH760463 | 1216 | _ | 58-945/295 |
|  | *G. mustelinum* | SSR44 | GDarSLA | Punjab-Multan | MH760464 | 1378 | _ | 70-1017/315 |
|  | *G. mustelinum* | SSR45 | AYVSA | Punjab-Multan | MH760465 | 1362 | _ | 57-944/295 |
|  | *G. mustelinum* | SSR46 | CLCuBuA | Punjab-Multan | MH760466 | 1368 | _ | 77-1024/315 |
|  | *G. mustelinum* | SSR47 | PaLCuA | Punjab-Multan | MH760467 | 1368 | _ | 73-1014/313 |
