# Supplementary figures and images for: Non-cultivated Cotton Species (Gossypium spp.) Act as a Reservoir for Cotton Leaf Curl Begomoviruses and Associated Satellites
Source: Plants (Basel). 2019 May 14;8(5):127. doi: 10.3390/plants8050127 (PMC6571856; doi:10.3390/plants8050127)

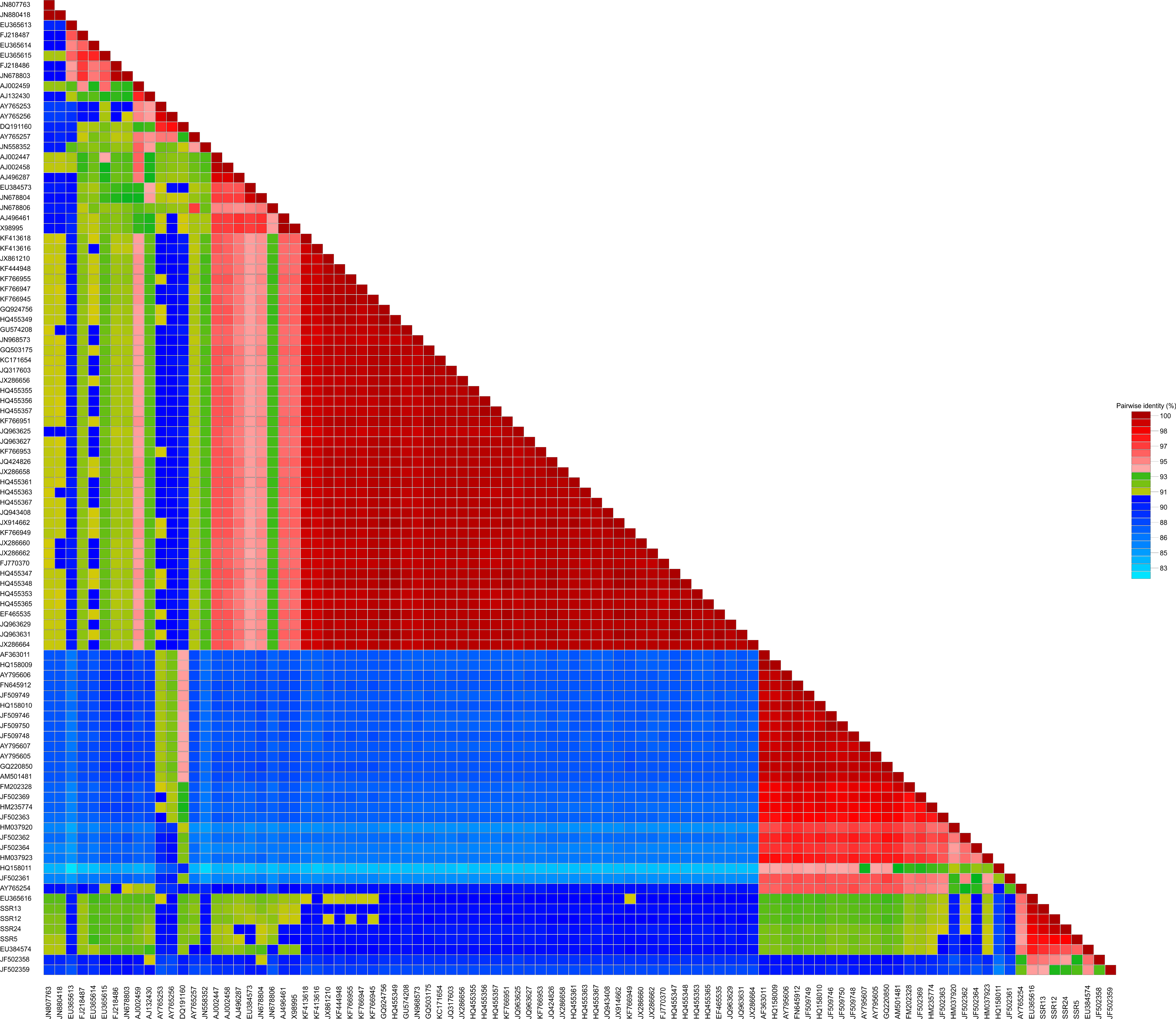

Supplement: Supplementary file 1 [file plants-08-00127-s001.zip › Supplementary Materials/Figure S1.jpg]

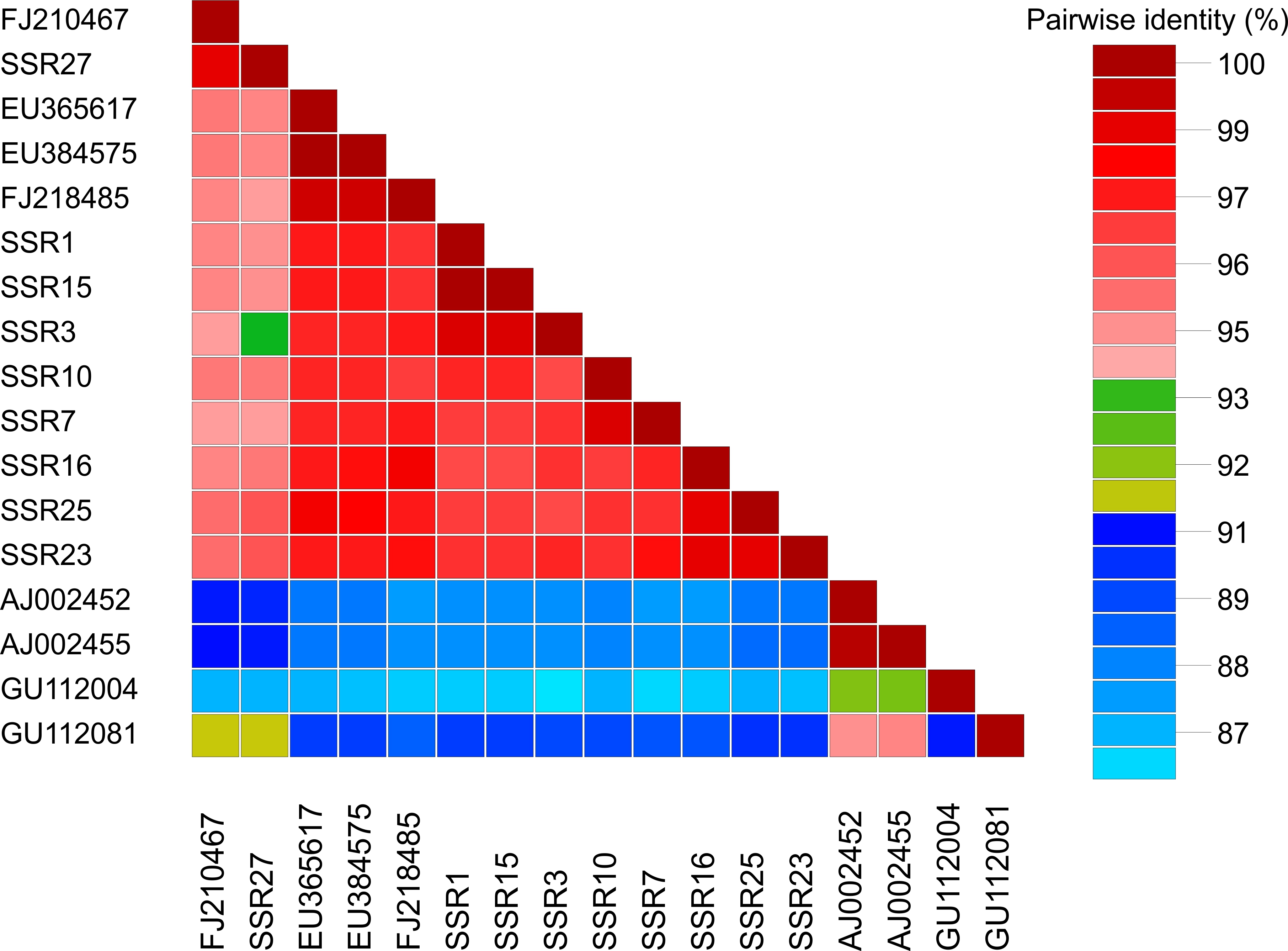

Supplement: Supplementary file 1 [file plants-08-00127-s001.zip › Supplementary Materials/Figure S2.jpg]

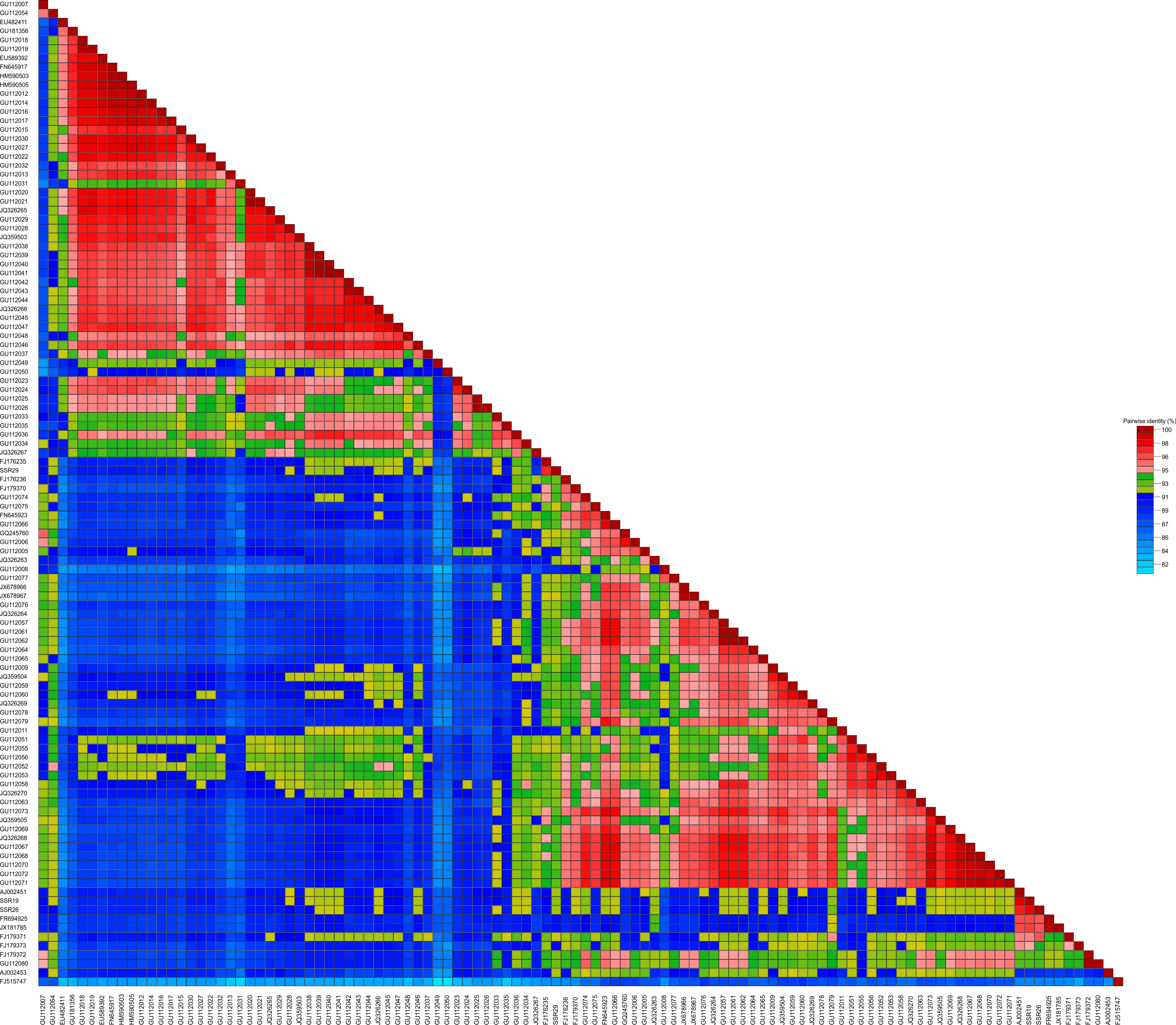

Supplement: Supplementary file 1 [file plants-08-00127-s001.zip › Supplementary Materials/Figure S3.jpg]

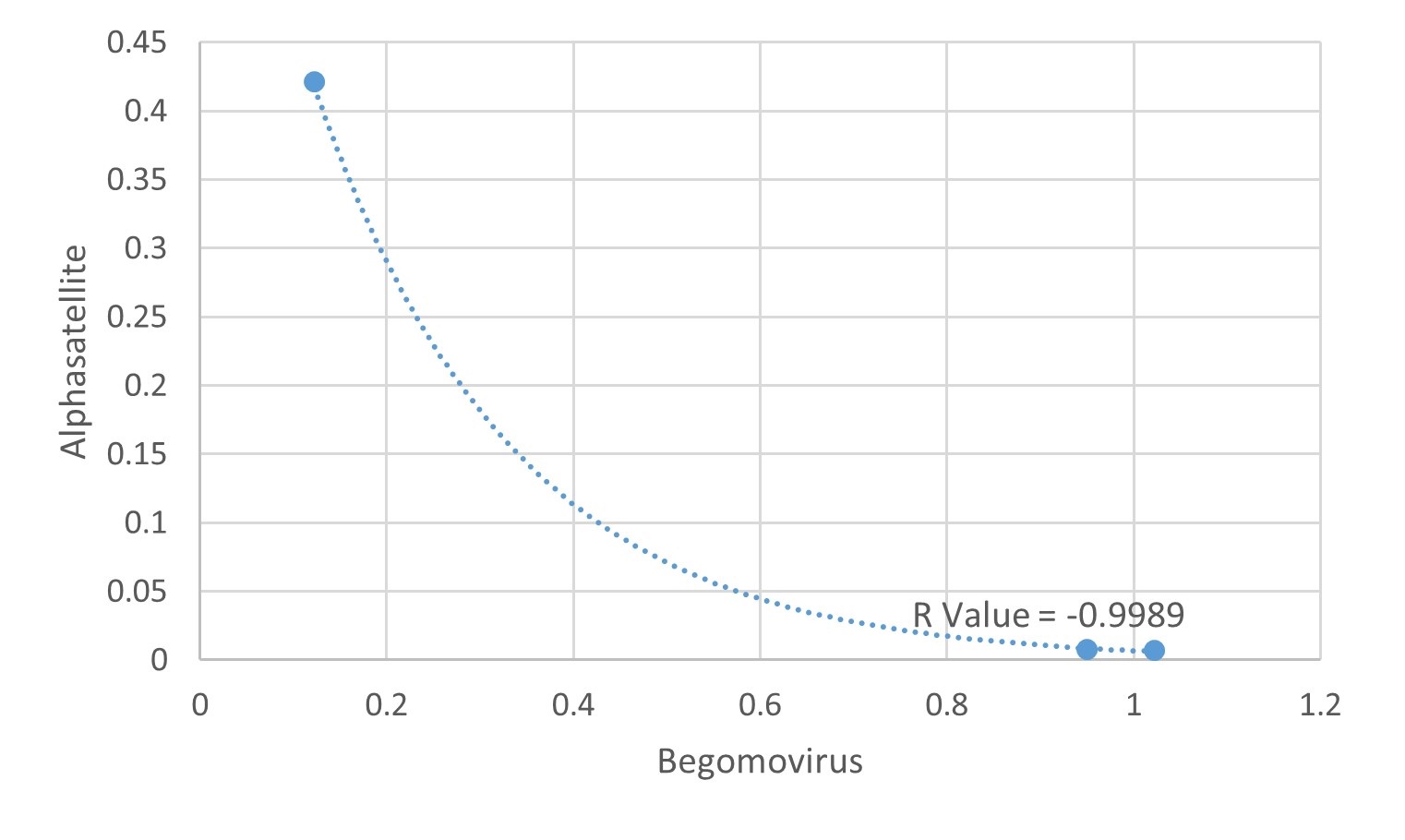

Supplement: Supplementary file 1 [file plants-08-00127-s001.zip › Supplementary Materials/Figure S4.jpg]
